# Anatomic Considerations for Radical Retropubic Prostatectomy in an Achondroplastic Dwarf

**DOI:** 10.1100/tsw.2009.24

**Published:** 2009-03-31

**Authors:** Dennis Gyomber, David Angus, Nathan Lawrentschuk

**Affiliations:** ^1^ Surgery and Urology, University of Melbourne, Studley Road, Heidelberg, Australia; ^2^ Department of Urology, University of Toronto, University Health Network, PMH, 610 University Avenue, 3-130, Toronto, ON, Canada

**Keywords:** prostate, neoplasm, prostatectomy, achondroplasia, anatomy

## Abstract

This is the first report of a radical retropubic prostatectomy (RRP) in an achondroplastic dwarf. We highlight the pelvic anatomy, precluding laparoscopic or robotic prostatectomy, and making open surgery extremely difficult. We review relevant literature regarding general, urological, and orthopedic abnormalities of achondroplasia (ACH) and present a clinical case. No reports of RRP in achondroplastic dwarfs exist, with only one case of an abandoned RRP due to similar pelvic anatomy in a patient with osteogenesis imperfecta. Significant lumbar lordosis found in ACH results in a short anteroposterior dimension, severely limiting access to the prostate. We present a case of a 62-year-old achondroplastic dwarf who had Gleason 3+4 disease on transrectal ultrasound-guided biopsy in four from 12 cores. Surgery was difficult due to narrow anteroposterior pelvic dimension, but achievable. Histological analysis revealed multifocal prostate cancer, with negative surgical margins and no extraprostatic extension. RRP in ACH patients, although possible, should be approached with caution due to the abnormal pelvic dimensions, and discussions regarding potential abandonment of surgery should be included during informed consent. This case highlights the preoperative use of computed tomography to assist in the surgical planning for patients with difficult pelvic anatomy.

## INTRODUCTION

This is the first report of radical retropubic prostatectomy (RRP) in an achondroplastic dwarf. Orthopedic abnormalities of achondroplasia (ACH) alter the pelvic anatomy such that access and exposure for RRP is extremely difficult. Such anatomical challenges dictate the type of approach necessary, and whether or not surgery is feasible. We highlight a case of significant prostate cancer treated with open RRP, and outline surgical and anesthetic considerations before proceeding in patients with ACH. In particular, the use of computed tomography for bony anatomy assessment was essential for surgical planning and would be of use in other cases of bony abnormality where RRP is being considered.

## CASE REPORT

A 62-year-old achondroplastic dwarf presented with moderate lower urinary tract symptoms. His digital rectal exam (DRE) was normal and he had a prostate specific antigen (PSA) of 4.1 ng/ml. Biopsy was offered, but he refused, and only agreed to it 1 year later when his PSA had risen (4.7 ng/ml) and his urinary symptoms worsened. A 12-core transrectal ultrasound-guided biopsy revealed Gleason 3+4 disease in >50% of four cores. Because of the unusual anatomy and risks of surgery, staging bone scan, plain radiography, and computed tomography (Fig. 1) were undertaken and were negative for metastases. Once the pelvic anatomy was examined, revealing a tiny anteroposterior diameter (3.6 cm) and distortion, but no other abnormality (Fig. 2), multidisciplinary review occurred.

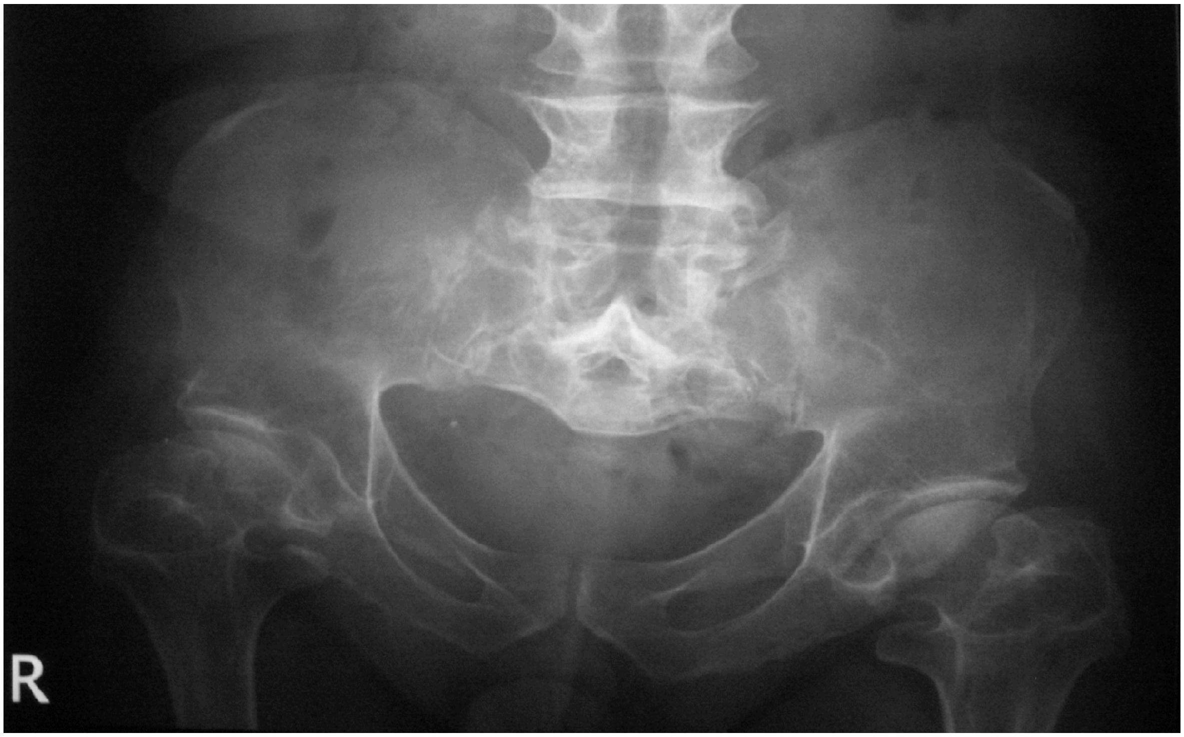

**FIGURE 1.** Plain abdominal radiograph in the ACH patient demonstrating an extremely narrow pelvis.

The outcome was that laparoscopic or robotic prostatectomy was inappropriate because of the lack of space. The patient had significant lower urinary tract symptoms, making brachytherapy unsuitable and radiotherapy challenging. Further, our radiation oncologists and physicists felt that planning for radiotherapy was difficult due to the bony anatomy and unusual shape of the pelvis, and the incomplete treatment could not be excluded. The patient was given external beam radiotherapy as an alternate option to surgery, but declined. We also considered focal therapy, high-intensity focused ultrasound, and cryoablation, but none were deemed appropriate or with enough long-term data[1,2].

This left an open RRP to be the most appropriate solution, although it was recognized that it would be extremely difficult with the prospect of pubectomy, if required. Open RRP was decided on by the patient after being given all options and, as expected, the surgery was difficult due to the narrow pelvic dimension, but achievable.

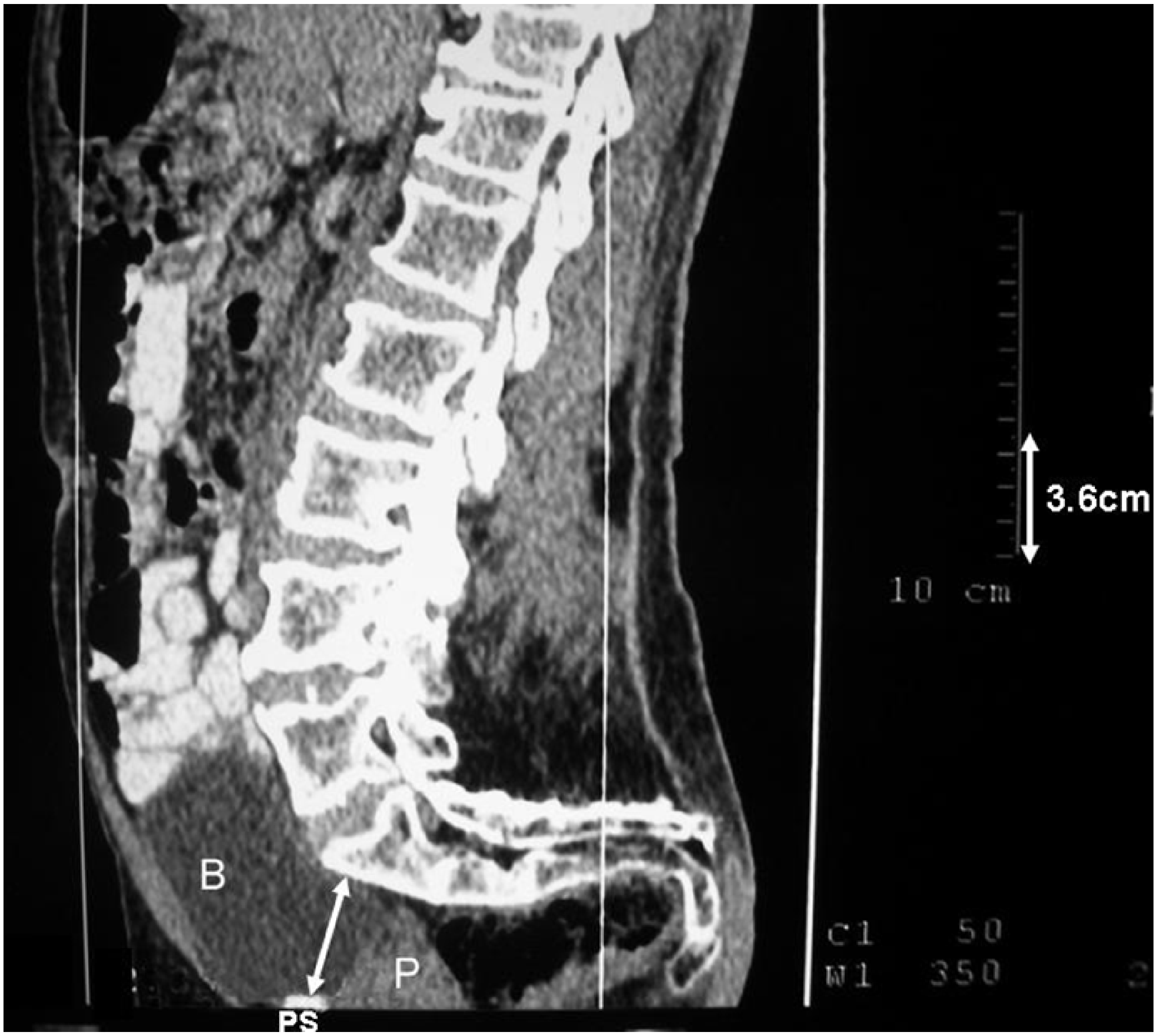

**FIGURE 2.** Saggital computed tomography scan section of the same ACH patient highlighting the bony anatomy, with the narrow anteroposterior dimension (arrow), and a distorted bladder (B) and prostate (P) behind the pubic symphysis (PS).

The patient was positioned in the supine position with legs slightly abducted. The operating table was broken with ten degrees of extension, hoping to open the narrow pelvis. Extra padding was required for his kyphotic spine to assist and prevent pressure areas, while extra lighting was used to assist with identifying structures. A lower midline abdominal incision was utilized to allow as much exposure vertically considering the narrow horizontal space. A Balfour self-retaining retractor with sponges as padding was used with a third blade to gain exposure, with the option of a Buckwalter that was not required. The dorsal venous complex was controlled by passing a McDougal forceps beneath the vein in a standard fashion and securing with 0-vicryl and then oversewing. A pubectomy was not required. Reasonable urethral length was obtained with retrograde dissection and ties/clips were used for the pedicles. Bilateral nerve sparing was undertaken. The longest available instruments were used with fine right angles borrowed for the procedure, with extra long handles to assist with nerve sparing. The bladder neck was opened anteriorly due to space limitations, with anastomotic suturing undertaken after bladder neck eversion, with the bladder more mobile than expected. Lymph nodes were not palpable and were not removed. Blood loss was minimal (350 cc); the patient was not transfused and operative time was reasonable (140 min).

Histological analysis revealed multifocal adenocarcinoma (Gleason 3+4), with negative surgical margins, no lymphovascular invasion[3], and no extraprostatic extension. His PSA at 15 months remains undetectable and he is continent with partial erections.

## DISCUSSION

ACH, known as short-limb dwarfism, is an autosomal-dominant condition involving mutation in the fibroblast growth factor receptor-3 (FGFR3) gene, with substitution of arginine for glycine[4]. The incidence is 17–26 per million and 80% are new mutations. The FGFR3 gene is responsible for bone ossification. Abnormalities of this protein product lead to abnormal ossification, resulting in the phenotypic appearance. These characteristics include short stature, short upper arms and legs, squared platypelloid pelvis, hyperlordosis, and macrocephaly. The hyperlordotic spine leads to an exaggerated lumbosacral lordosis, causing forward rotation of the pelvis, creating the prominent abdomen and buttocks[4].

Associated health problems for consideration for anesthetics include cardiac and respiratory difficulties, recurrent ear infections, and cervical vertebral instability[4,5]. Hence, ACH dwarfs have increased mortality compared to the normal population, but they are living longer than in the past[5] and, therefore, are more at risk from prostate cancer. The urology literature needs to have examples to help guide treatment decisions and surgical planning. The literature does not report any association between ACH and prostate cancer, however, a mutation in the FGFR3 gene has been found in bladder cancer and there may be a theoretical increased bladder cancer risk in this population[6]. This is of interest as it is understood that an increased risk of bladder cancer confers an increased risk of prostate cancer[7].

Pelvimetry is the radiological measurement of the pelvic bony dimensions[8]. Interestingly, where pelvic dimensions are narrower than average, there has been an association with a greater likelihood of capsular incision in RRP, but not operative morbidity[9]. This is difficult to translate to ACH cases as anteroposterior dimensions may be extreme (as in this case, 3.6 cm) compared to an average male (>10 cm)[9]. Excluding obstetric issues, there have been no reports highlighting the difficulties of pelvic surgery in achondroplastic dwarfs secondary to abnormal pelvic anatomy. However, difficult pelvic anatomy has been reported as a reason for abandoning planned radical prostatectomy[10]. In the past, alternative approaches for difficult bony anatomy and access for RRP included total and partial pubectomy[11,12] and such measures need to be considered when the pelvis is narrow.

In this case, we felt patient positioning to be critical and extension essential to open the pelvis as much as we could. The principles of exposure, lighting, and adequate instrumentation (in this case extended instruments) were essential. Due to our experience using lower midline incisions, with the potential to extend vertically combined with the narrow width of the pelvis, we felt more comfortable than using a horizontal incision (e.g., Cherney or Pfannenstiel), which we concede could also have been used.

In conclusion, pelvic surgery in ACH patients should be approached with caution. Multidisciplinary discussions regarding possible alternative forms of management and, where surgery is chosen, pubectomy or partial pubectomy and the possibility of unsuccessful surgery should all be considered. This case highlights the preoperative use of computed tomography to assist surgical planning in patients with difficult pelvic anatomy.
